# A sea anemone of many names: a review of the taxonomy and distribution of the invasive actiniarian *Diadumene lineata* (Diadumenidae), with records of its reappearance on the Texas coast

**DOI:** 10.3897/zookeys.706.19848

**Published:** 2017-10-04

**Authors:** Zachary B. Hancock, Janelle A. Goeke, Mary K. Wicksten

**Affiliations:** 1 304 Butler Hall, 3258 Texas A&M University, College Station, TX 77843-3258, USA; 2 3029 Texas A&M University, Galveston, TX 77554-2853, USA

**Keywords:** Galveston Island, Gulf of Mexico, invasive species, salt marsh, Texas coast

## Abstract

*Diadumene lineata* (Actiniaria: Diadumenidae) is a prolific invader of coastal environments around the world. First described from Asia, this sea anemone has only been reported once from the western Gulf of Mexico at Port Aransas, Texas. No subsequent sampling has located this species at this locality. The first record of the reappearance of *D. lineata* on the Texas coast from three locations in the Galveston Bay area is provided, and its geographic distribution and taxonomic history reviewed.

## Introduction

*Diadumene lineata* (Verrill, 1869) is perhaps the most widespread actiniarian in the world (Seaton 1985, Fautin et al. 2009a). Believed to be native to Japan or Hong Kong (Verrill 1869, Uchida 1932) where it was first described by the American naturalist A.E. Verrill, it has since been reported from almost every coast. The sea anemone was discovered in North America at Woods Hole, Massachusetts in 1892 by Verrill’s daughter, Lucy. He wrote in his description, “My attention was first called to this species… by my young daughter, Miss Lucy L. Verrill, for whom I have named it” (Verrill 1898). Not realizing that the anemone was the same species he had described from Hong Kong (*Sagartia lineata*), he named it *Sagartia luciae*. Parker (1902) extended the known range of *D. lineata* in New England to Rhode Island, and by 1929 it had been collected in Cape Charles, Virginia (Richards 1931). Gosner (1971), in his key to invertebrates from Cape Hattarus to the Bay of Fundy, reported this animal occurred as far south as North Carolina. Calder and Hester (1978) included *D. lineata* (as *H. luciae*) in a checklist of actiniarians from South Carolina, describing them as “common to abundant.” On the Pacific coast, Torrey (1904) described *Sagartia davisi* from San Pedro, California, now considered a synonym for *D. lineata*. The species was included in a faunal checklist of California as early as Johnson and Snook (1927), who wrote “*Sagartia luciae*… has been reported from San Francisco. We have found this similar form to be very common at certain points in San Diego Bay and Mission Bay.” Cutress (1949) reported this sea anemone occurred on the Oregon coast and Morris et al. (1980) extended the range to Washington, expanding its occurrence to the entire US Pacific coast. Hand (1964) reported its first occurrence in Britain as 1896; however, if *Chrysoela chrysosplenium* was originally composed of both itself and *D. lineata* as suggested by Fautin (2015), it was found in Cornwall as early as 1847. Cornelius et al. (1995) included the species as *H. lineata* in their guide to European marine fauna, and wrote that it occurred on, “all British coasts… widely distributed through Europe and the rest of the world” (Cornelius et al. 1995). Ocaña and Den Hartog (2002) included *D. lineata* from the Canary Islands off the coast of North Africa, and Zabin et al. (2004) reported the first occurrence of the anemone on the Hawaiian Islands. It was first recorded from India by Parulekar (1968) in a list of Actiniaria of Bombay. Fautin et al. (2009b) added *D. lineata* in their checklist of actiniarians known to be common on the coast of Singapore, and Cha and Song (2001) report the species occurred on Jeju Island of South Korea by 1985. Liu et al. (2003), in a review of the diversity of sea anemones of Lianyun Harbor in the Jiangsu Province of China, included *Haliplanella luciae* in their species list. Along the southwest Atlantic coast of South America, the anemone was first reported from Rio de Janeiro, Brazil by Belem and Monteiro (1977), and found at the busy Port of Recife by Farrapeira et al. (2007) as part of a study of fouling organisms on ship hulls. In the Argentine Sea, *D. lineata* was first reported by Excoffon et al. (2004). *Diadumene lineata* was not reported from the South Pacific coast until 2015, when it was found at Coquimbo, in northern Chile (Häussermann et al. 2015).

There exist only two reports of *D. lineata* in the Gulf of Mexico, most recently by Minasian and Marsical (1979) from northwestern Florida. The earlier record is from Port Aransas, Texas at the University of Texas Marine Research Station (Carlgren and Hedgpeth 1952). Hedgpeth (1954) included *D. lineata* (as *Aiptasiomorpha luciae*) in the checklist of cnidarians of the Gulf of Mexico, citing Carlgren and Hedgpeth (1952) as the only known record from west of Florida. However, *D. lineata* has not been found at Port Aransas since (Wicksten, pers. obs.). Additionally, to our knowledge no other reports of *D. lineata* exist in the Gulf west of northern Florida.

**Figure 1.**
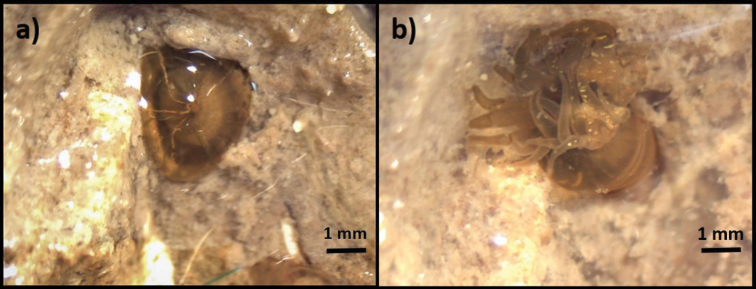
*Diadumene lineata* at Sportsman Road on Galveston Island with tentacles fully retracted (**a**), and with tentacles extended (**b**).

The small (5–10 mm in diameter), often inconspicuous sea anemone is dark green or brown with orange, yellow, white, or green vertical stripes (Ruppert and Fox 1988) and resembles a gelatinous peppermint (Figure 1). *Diadumene lineata* occurs in dense numbers on rock jetties, pilings, oyster reefs, and in salt marshes where it has been reported to associate with *Spartina alterniflora* (Molina et al. 2009). *Diadumene lineata*’s incredible potential for invasion has been attributed to asexual reproduction via longitudinal fission or pedal laceration (Ting and Geller 2000) and the tendency for larvae to adhere to boat hulls (Gollasch and Riemann-Zürneck 1996). Johnson and Snook (1927) note that when submerged in a bucket of seawater, “[the sea anemone] will move to the bottom or sides… and begin dividing without delay.” Additionally, *D. lineata* can survive and reproduce in a broad range of conditions. The species can tolerate salinities ranging from over 35 ppt down to 5 ppt, or even lower at cold temperatures, and can withstand temperatures from 0 °C to at least 27.5 °C (Shick 1976). It can also withstand moderate levels of hypoxia (Jewett et al. 2005). The ability to survive in such diverse and sometimes harsh conditions has contributed to *D. lineata*’s ability to spread and survive.

## Materials and methods

**Figure 2.**
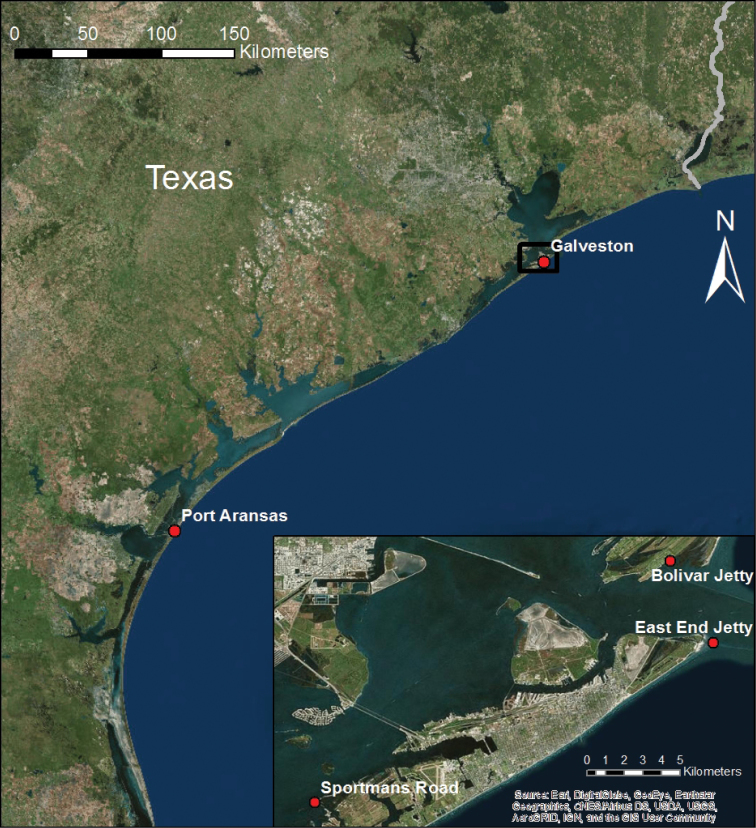
Map showing the Texas Coast with Port Aransas and Galveston marked. Inset shows the three locations around Galveston Bay where *D. lineata* populations were found: East Beach (29°19.9'N; 94°43.5'W), Bolivar jetty (29°22.2'N; 94°45.0'W), and Sportsman Road (29°15.2'N; 94°55.1'W).

**Figure 3.**
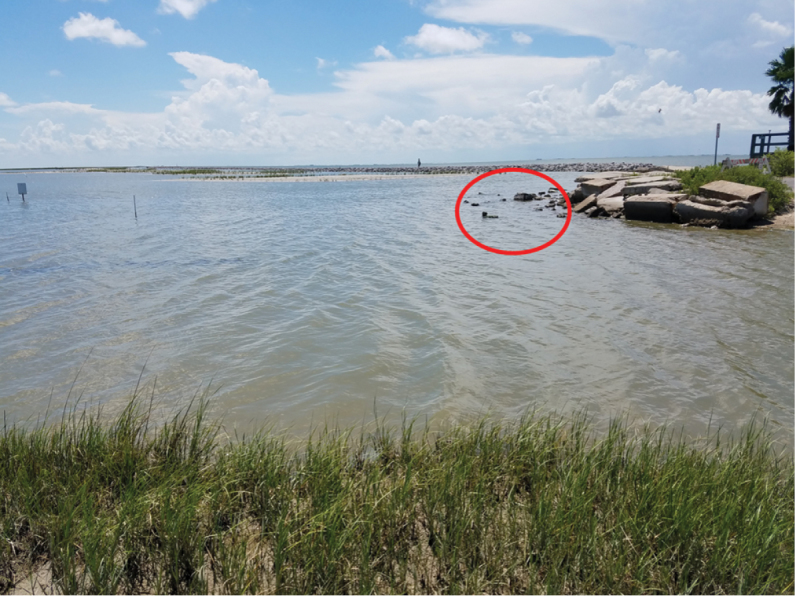
A view of the Sportsman Road site. The circled area indicates where anemones were sampled from in this study; anemones have also previously been located on the jetty in the background (Wicksten, pers. obs.).

To date, *D. lineata* has been discovered at three separate locations in the Galveston Bay area (Figure 2). *D. lineata* was first discovered on Galveston Island in November 2016 on a jetty at East Beach (29°19.9'N, 94°43.5'W) (Sutherland, personal communication). Another population was discovered on a rock jetty that supports a *Crassostrea virginica* Gmelin reef on Bolivar Peninsula on March 14, 2017 (29°22.2'N, 94°45.0'W). Five individuals were gently scraped from the oyster shells and preserved in 70% ethanol. A third population was discovered on a bed of oyster shells near a salt marsh off Sportsman Road on Galveston Island on July 7, 2017 (29°15.2'N, 94°55.1'W) (Figure 3). Individuals from the Sportsman Road population were collected and photographed with a Moticam 10 microscope camera, and subsequently stored in 70% ethanol. These individuals were small, less than 5 mm in diameter, and were located by removing several oysters and bringing them back to the laboratory for observation under a dissecting microscope. Additional specimens have been collected and stored in formalin.

As the anemones were dark green with the characteristic orange or white stripes, they were readily diagnosed as *D. lineata*. This is one of the few species of anemones that is unmistakable by color pattern (Fautin et al. 2009a).

### Taxonomic review of *Diadumene lineata*

Hand (1964) wrote concerning *D. lineata*, “This common and well known species has a very discouraging synonymy…” A list of all synonymized names from the World Register of Marine Species (WoRMS) includes various names (Fautin 2015; Table 1). The taxonomic history of *D. lineata* has been plagued with confusion since the beginning.

**Table 1.** Synonyms, misidentifications, and their localities for *D. lineata* (after Fautin 2015).

Taxonomic identification	Locality	Source
*Actinia chrysosplenium*	St. Ives, Cornwall	Johnston 1847
Actinea chrysoplinum*	Falmouth, Cornwall	Cocks 1850
Actinea chrysosplenum*	Falmouth, Cornwall	Cocks 1851
*Bunodes chrysosplenium*	Britain	Gosse 1855
*Sagartia chrysosplenium*	Cornwall	Gosse 1860
*Chrysoela chrysosplenium*	Cornwall	Gosse 1860
*Sagartia lineata*	Hong Kong	Verrill 1869
Sagartia chrysosplenium*	Britain	Pennington 1885
*Sagartia pustulata*	North Carolina	McMurrich 1887
*Sagartia luciae*	Woods Hole, Massachusetts	Verrill 1898
*Sagartia davisi*	San Pedro, California	Torrey 1904
*Diadumene luciae*	Britain	Stephenson 1925
*Aiptasiomorpha luciae*	Oregon	Cutress 1949
*Haliplanella luciae*	California	Hand 1955
Haliplanela luciae*	France	Dominique et al. 1985
*Diadumene lineata*	Wells-next-the-sea, Norfolk	Williams 1980
Haliplanella lucia*	Korean Strait	Song 1992
Haliphlanella luciae*	N/A	Hand and Uhlinger 1994
*Haliplanella lineata*	Europe	Cornelius et al. 1995
Haliplannella luciae*	N/A	Grosholz and Ruiz 1996
Haliplanella liciae*	China	Pei 1998

*Indicates misspellings, not unique taxonomic identifications

Verrill (1871) described *Sagartia lineata*, a species he stated to be “common on stones and pebbles among gravel” that had been collected by Dr. William Stimpson on the Hong Kong harbor. McMurrich (1887) soon after described *S. pustulata* from Beaumont, North Carolina, which is included as a synonym for both *D. lineata* and *Actinothoe pustulata* (Carlgren 1949; Hand 1955; Fautin 2015). Later, Verrill (1898), unsuspecting that *S. lineata* could occur in both Hong Kong and Woods Hole, erected *Sagartia luciae*. As stated in the Introduction, Torrey (1904) described what he thought a new species of *Sagartia*, *S. davisi*, from California. Stephenson (1925) suggested reorganizing *S. luciae* to the genus *Diadumene* after McMurrich (1921) presented anatomical evidence suggesting the species did not belong in the genus *Sagartia* (for example, McMurrich notes six pairs of mesentery and the lack of a sphincter).

McMurrich (1921) considered *S. luciae* to be synonymous with *S. chrysosplenium* (Cocks, 1847). Johnston (1847), citing an unpublished description by W.P. Cocks, described a small anemone that was “bright pea-green to dark holly-leaf tint, stripped or dotted with bright yellow” that occurred in St. Ives, Cornwall. Cocks himself would later include the anemone, which he called *Actinia chrysosplenium*, in two later works from Falmouth (Cocks 1850, 1851). Goess (1855) moved the anemone to the newly erected genus *Bunodes* as he at the time believed the surface was “warty” after examining plates that appeared in Johnston (1847). However, Gosse (1860), quoting a correspondence with W.P. Cocks, stated, “…when I examined the body of *chrysosplenium* with a lens of two inches’ focus… in appearance resembled a piece of smooth Indian-rubber… not the slightest trace of tubercles apparent,” leading Gosse to reclassify the anemone to the genus *Sagartia*. Even in this assignment Gosse was uncertain, and suggested placing the species in a new genus, which he called *Chrysoela*, meaning “that which is studded with golden nails” (Gosse 1860).

While McMurrich (1921) considered *S. luciae* and *S. chrysosplenium* (=*Chrysoela chrysosplenium*) to be synonymous, Stephenson (1925) argued that the two species were distinct, finally fully separating *S. lineata-luciae* from *C. chrysosplenium*, a separation that exists to this day. Hand (1955), in a review of the anemone’s synonymy, concluded that the relationship between *D. lineata* and *C. chrysosplenium* could not be determined as an actiniarian matching Cocks’ original description had not been sampled. Fautin (2015) includes *C. chrysosplenium* both as a synonym for *D. lineata* and as a separate species.

McMurrich (1921) offered the first suggestion that *S. lineata* and *S. luciae* were the same species. Calgren (1949) listed *D. lineata* as *Aiptasiomorpha luciae*, which would be adopted by Cutress (1949) in the description of anemones on the Oregon coast and Carlgren and Hedgpeth (1952) in the record of *D. lineata* at Port Aransas. Hand (1955) combined *Aiptasiomorpha* and *Diadumene*, arguing that the only distinguishing feature was the presence of catch tentacles in the latter, but not all individuals of the Diadumenidae possessed them. She further erected the family Haliplanellidae with a single genus *Haliplanella* on the basis of a novel combination of nematocysts in the acontia of basitrichs, microbasic p-mastigophores, and microbasic amastigophores, and removed *D. luciae* to this new genus. However, the genus *Haliplanella* had previously been established by Treadwell (1943) for a group of polychaetes, thus rendering it invalid. The invalid name *Haliplanella luciae* still appears in many modern texts (Calder and Hester 1978; Morris et al. 1980; Ruppert and Fox 1988; Ruppert et al. 2004).

**Figure 4.**
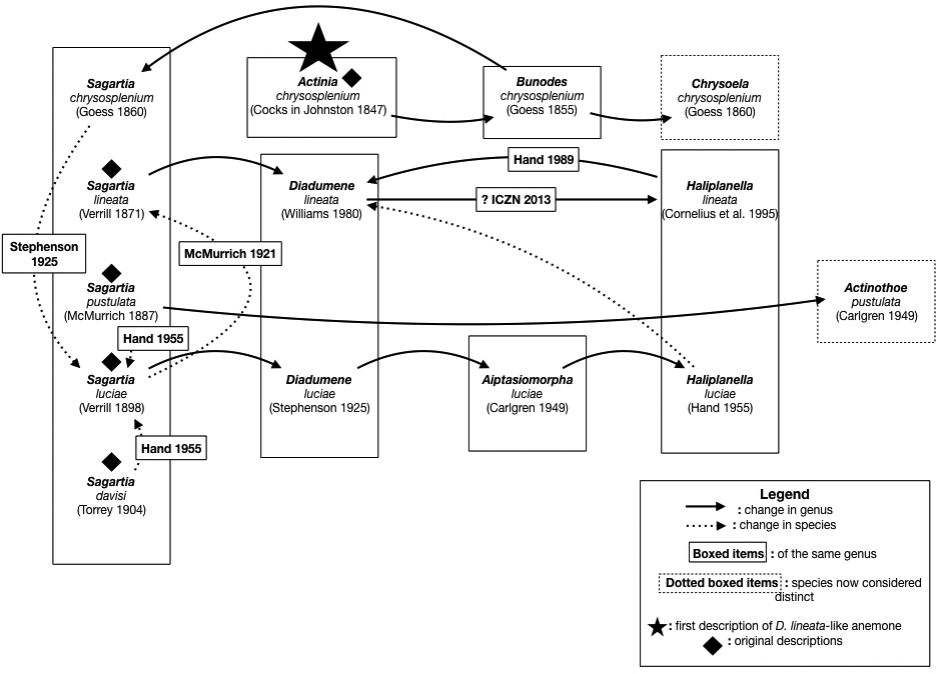
Synonymy flowchart, including misidentifications (i.e., *Chrysoela chrysosplenium* and *Actinothoe pustulata*). Synonyms based on misspellings have been omitted.

**Figure 5.**
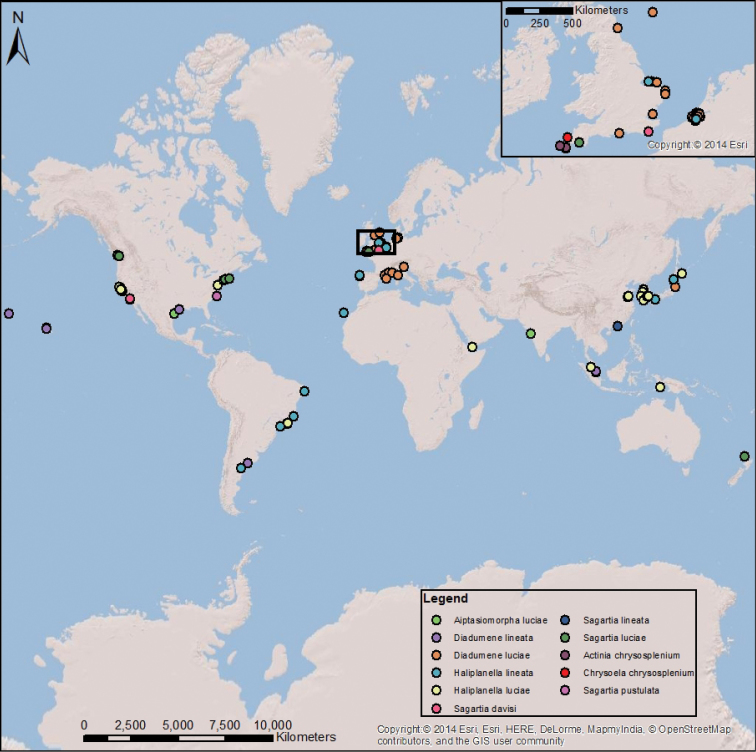
A map showing name usage by location for the synonyms of *D. lineata*, not including misspelled names. Inset shows Britain and the northern coast of Europe where there is a very high density of references (adapted from Fautin 2015).

**Figure 6.**
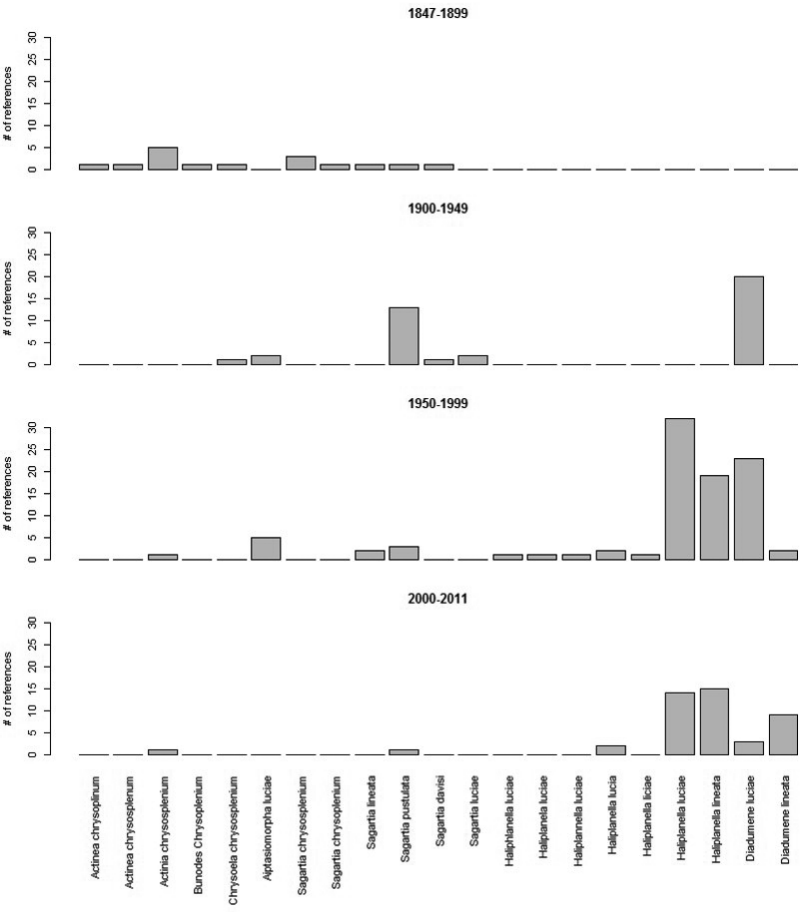
Histogram showing the shift in name usage over time. In the time period 2000-2011, *Diadumene lineata* showed the greatest increase in usage. Data compiled from Fautin (2015).

Hand (1989) conceded the anemone to the genus established by Stephenson (1925), and recognizing Verrill’s original description the name *Diadumene lineata* was established. Despite this, Fautin and Hand (1977) and Fautin et al. (2009a) petitioned the International Commission of Zoological Nomenclature (ICZN) to preserve the genus *Haliplanella* by suppression of Treadwell (1943), and were rendered an affirmative opinion by the commission (ICZN 2013). Fautin et al. (2009a) acknowledged the wide acceptance of Verrill’s original description of *S. luciae* to be a synonym for *S. lineata* (Seaton 1985), and proposed the proper name to instead be *Haliplanella lineata*. Given the commission’s opinion that the genus name *Haliplanella* be accepted, writing that it is, “conserved for a widespread sea anemone,” (i.e. *D. lineata*), confusion as to the generic position of *D. lineata* remains. Several recent publications mention the name *Haliplanella lineata* (e.g. Goulletquer et al. 2002; Molnar et al. 2008), but at present, the name *Diadumene lineata* is considered valid (Cairns et al, 2002; Fautin 2015). A flowchart is presented here depicting taxonomic reorganizations of *D. lineata*, including all known synonyms and misidentifications, excluding misspellings (Figure 4). A map of *D. lineata*’s range indicates the first occurrences at each respective location (Figure 5). We have adopted the binomen *Diadumene lineata* here on the basis that the most recent studies of the sea anemone have used this name (Ting and Geller 2000; Cairns et al. 2002; Molina et al. 2009; Podbielski et al. 2016). The shifts in usage of *D. lineata* synonyms in publications through time are presented in a histogram (Figure 6).

To our knowledge, no comprehensive molecular study exists suggesting that *D. lineata* is a species complex instead of a single, worldwide species. However, *D. lineata* populations do harbor greater genetic diversity than had been previously thought (Ting and Geller 2000).

### Associated species

Organisms found in association with *D. lineata* were those typical of Texas coast oyster reefs, including corophiid amphipods, an array of polychaete worms, nemerteans, cheilostomate bryozoans, xanthid crabs, the anomurans *Clibanarius vittatus* Dana and *Petrolisthes armatus* Gibbes, and the solitary ascidian, *Molgula* sp.

## Discussion

While *D. lineata* has been reported as cosmopolitan (Cornelius et al. 1995, Fautin and Daly 2009), its occurrence in the western Gulf of Mexico has been uncertain since Carlgren and Hedgpeth (1952). All references to *D. lineata* as being present in the western Gulf rely on this single citation (Fautin and Daly 2009). While we do not dispute that the anemone appeared in Port Aransas in 1952, it is likely to have not become established, suffering local extinction. This is not uncommon for *D. lineata* invasions (Ruppert and Fox 1988). As this anemone is small, the possibility exists that it has been overlooked; however, we consider this unlikely as regular sampling trips to Port Aransas have been carried out since 1990 as part of the Texas A&M University Marine Biology field trip, which includes transects of various rock jetties, and have not located it. After Carlgren and Hedgpeth (1952) there is no record of any search for the anemone in Port Aransas until the 1980s (Wicksten, personal observation). Therefore, it is impossible to know when in that time period the anemone disappeared. Without this information we are unable to establish an exact cause of extinction. Possible causes include unusually high summer temperatures in one or more years heating the shallow waters the anemones inhabit above their survival range, or an inability to establish a stable population due to the relatively small amount of foreign shipping at Port Aransas introducing few individuals.

Therefore, based on the assumed disappearance of the Port Aransas population, the current report represents a reappearance of *D. lineata* on the Texas coast, and the first established population in the western Gulf of Mexico to the best of our knowledge. Other locations, including Christmas Bay (29°53.4'N, 95°6.9'W>) and the Fin and Feather Reef on Redfish Bay (27°53.4'N, 97°6.9'W), have been examined in the past, but no sea anemones were found (Wicksten, personal observation). Based on the three separate observations of the Galveston Bay *D. lineata* populations, which occurred across a range of locations and dates, we are confident that *D. lineata* is well established in the bay. Salt marshes near the Sportsman Road jetty were examined for any association between *D. lineata* and *S. alterniflora* as reported by Molina et al. (2009), but no sea anemones were found. This could reflect that the association between *D. lineata* and *S. alterniflora* is limited to the Bahia Blanca estuary, or simply the difficulty of locating such tiny animals in the dense muddy marsh without the use of cores and laboratory observation.

It is unsurprising that *D. lineata* became established in Galveston Bay of all locations in the western Gulf. An enormous amount of cargo passes through Galveston Bay every year, as passage through the Bay is required to reach not only the Port of Galveston, but also the much larger ports of Houston and Texas City. In 2015 the American Association of Port Authorities ranked the ports of Houston and Texas City 1st and 15th respectively in terms of the total tons of annual foreign imports (American Association of Port Authorities 2015). The port of Galveston itself includes regular shipping lines for vessels that dock in areas known to have been invaded by *D. lineata*. For example, American Roll-On Roll-Off Carrier (ARC) includes a trade route that connects Galveston to Southampton, roughly 150 km from Cornwall where the species has been located previously. Höegh Autoliners, another Galveston port regular, ports extensively through Southeast Asia where *D. lineata* is believed to originate. Past studies have found this species adhering to boat hulls and suggested this as a likely explanation for its worldwide invasion (e.g., Gollasch and Riemann-Zürneck 1996; Farrapeira et al. 2007).

The generic position of *D. lineata* still appears in dispute, as the taxonomic review above indicated, while the specific name *D. lineata* seems accepted due to precedent (Fautin et al. 2009a; Fautin 2015). The 2013 ICZN decision to suppress Treadwell (1943) does not appear to resolve the generic position; however, the tide seems to have shifted in favor of *Diadumene* as the proper genus (Ting and Geller 2000; Molina et al. 2009; Fautin 2013; Podbielski et al. 2016).
